# Surveillance of Landraces’ Seed Health in South Italy and New Evidence on Crop Diseases

**DOI:** 10.3390/plants12040812

**Published:** 2023-02-11

**Authors:** Eliana Dell’Olmo, Massimo Zaccardelli, Vincenzo Onofaro Sanaja, Boris Basile, Loredana Sigillo

**Affiliations:** 1 Council for Agricultural Research and Economics, Research Centre for Vegetable and Ornamental Crops, Via Cavalleggeri 25, 84098 Pontecagnano, Italy; 2 Department of Agricultural Sciences, University of Naples Federico II, 80055 Portici, Italy

**Keywords:** landraces, legumes, pathogens, *Apiospora arundinis*, *Sclerotinia sclerotiorum*, *Stemphylium vesicarium*, phytosanitary screening

## Abstract

During the last three years, more than 300 landraces belonging to different plant species have been the main focus of an Italian valorization research project (AgroBiodiversità Campana, ABC) aiming at analyzing, recovering, preserving, and collecting local biodiversity. In this context, phytosanitary investigation plays a key role in identifying potential threats to the preservation of healthy seeds in gene banks and the successful cultivation of landraces. The surveillance carried out in this study, in addition to highlighting the expected presence of common species-specific pathogens such as *Ascochyta pisi* in peas, *Ascochyta fabae* in broad beans, and *Macrophomina phaseolina*, *Xanthomonas axonopodis* pv. *phaseoli*, and *Xanthomonas fuscans* subsp. *fuscans* in beans, pointed to the presence of novel microorganisms never detected before in the seeds of some hosts (*Apiospora arundinis* in common beans or *Sclerotinia sclerotiorum* and *Stemphylium vesicarium* in broad beans). These novel seedborne pathogens were fully characterized by (i) studying their morphology, (ii) identifying them by molecular methods, and (iii) studying their impact on adult crop plants. For the first time, this study provides key information about three novel seedborne pathogens that can be used to correctly diagnose their presence in seed lots, helping prevent the outbreaks of new diseases in the field.

## 1. Introduction

Nowadays, most agricultural systems are based on the cultivation of commercial varieties that assure high mass yields, seriously limiting the preservation of biodiversity [1]. Much concern of the scientific community about ongoing biodiversity erosion derives from the observation that 70% of biodiversity has been lost during the last century and, even more alarmingly, most of the food consumed worldwide is produced from only 12 vegetable species [2,3,4,5]. This dramatic scenario has forced governments to act at a global and local scale to define strategies aiming to discover and preserve biodiversity [6,7]. With this aim, an increasing importance has been given recently to landraces as a tool to preserve agrobiodiversity. The landraces are defined as dynamic populations characterized by phenotypical diversity, genetic diversity, and adaptative features closely related to the environment where they have been developed [8,9]. Landraces represent an important resource because they are adapted to low-input agriculture and are suitable for cultivation in harsh environments [10]. The high specialization of landraces provides a pool of genes that can be used in plant breeding to increase the resistance of cultivated crops to abiotic stressors such as drought and floods, and to biotic stressors such as pests and diseases [3]. In addition, landraces play a key economic role in low-income countries, while in high-income nations they are considered as niche products [8,11], being perceived by the consumers as high-quality products [11]. Moreover, landraces were part of the cultural heritage of territories and were used in traditional food and local festivals [9]. Therefore, landraces are gaining importance due to their pivotal role in the cultural heritage of populations and their interesting properties, such as stability, adaptability, and nutritional values [4,8,9]. In order to preserve biodiversity and enhance knowledge about landraces, the global community has established guidelines such as the European Union Biodiversity Strategy to 2020 and the 2030 Agenda for Sustainable Development [6,12,13]. Accordingly, Italy established a national system for the conservation and enhancement of biodiversity with law n. 194 of 1 December 2015, which aimed at protecting local genetic resources from the risk of extinction and/or genetic erosion [6].

The increasing interest in landraces has led to the definition of international programs dedicated to their valorization and inclusion in germplasm banks [5], where plant material needs to be stored in perfect health conditions. One of the major issues linked to landrace conservation is the self-production of the seeds by local farmers, which often results in low-quality propagation material because of the presence of seedborne pathogens. Seeds are considered the first vehicle of pathogens in the environment and, in the worst case, they can introduce novel pathogens in a habitat or cause the re-emergence of diseases previously eradicated [14]. The successful storage of seeds in germplasm banks requires integrated seed health strategies that were developed all over the world [6] to mitigate the impact of seedborne pathogens on crops, and are based on the employment of secure propagation materials. In this context, the use of seeds that are certified disease-free or with a concentration of pathogens below imposed thresholds may represent a primary strategy to counteract seedborne diseases [15,16]. For this reason, there is an urgent need for the setup of methodologies aiming to provide diagnostic tools that are effective in detecting the complexity of pathogenic microflora. Knowledge of the biological cycles of the emerging pathogens and their aptitude for transmission from seeds to plants must also be sought to evaluate the real risk of epidemics [17].

The aim of the present work was to contribute to the knowledge about pathogenic microflora resident on seeds of Fabaceae, Brassicaceae, and Liliaceae landraces in order to provide new insights about the risks of losing biodiversity and the spread of seedborne disease outbreaks. Furthermore, this approach is oriented to (i) ensure the optimal phytosanitary conditions of seeds to be used as propagation materials, (ii) contribute to disease control measures, and (iii) limit the spread of pathogens in the environment. The research activity described in this work was carried out as part of the phytosanitary surveillance of the seed health of landraces that originated in the Campania region (Southern Italy) and are included on the priority list considered in the national project on biodiversity, “Agro Biodiversità Campana” (ABC), whose major aim was to analyze, recover, preserve, and collect local biodiversity. The results reported here complement those previously described for the fungi belonging to the Botryosphaeriaceae family associated with accessions of common bean (*Phaseolus vulgaris* L.) [18,19].

## 2. Results

### 2.1. Liliaceous Crops: Garlic (Allium sativum L.) and Onion (Allium cepa L.)

In this work, two accessions of garlic, “Bianco locale” and “Ecotipo locale di Caposele”(Table 1), were subjected to mycological screening on the cloves, which are the most commonly used propagation material for this crop. After seven days of plate incubation, fluffy, pink, *Fusarium*-like colonies developed from tissue fragments of “Bianco locale”. DNA extracted from a representative monoconidial culture (CREA OF 1119.1) was amplified in the ITS1 region. Using the primers listed in Table 2, the obtained sequence OQ148577 was aligned in GenBank, and the isolate was recognized as *Fusarium proliferatum* (Matsush. Nirenberg ex Gerlach & Nirenberg) after matching with the accession MK252904 of isolate PHVNO21 of *F. proliferatum* (Table 3).

Regarding three onion landraces (“Di Alife”, “Agostina”, and “di Eremiti”), screened for the presence of pathogenic fungi, the results showed that the only contamination was caused by the saprophytic *Alternaria* spp.. The BLASTn analyses performed with the ITS sequence identified the CREA OF 1115.1 (OQ280977) strain as *A. alternata* with an identity of 97.80% with strain SS_13 (MT497390) (Table 2).

### 2.2. Leguminous Crops and Novel Pathogen Reports

#### 2.2.1. Cowpea (*Vigna unguiculata* (L.) Walp) and Common Bean (*Phaseolus vulgaris* L.)

The analyses carried out on V. unguiculata did not reveal the presence of fungal or bacterial pathogens. Conversely, all three accessions (“Corna dei Signori (San Marzano)”, “Fagiolino lungo” (San Marzano), and “Corna dei Signori” Caste S. Giorgio) resulted positive for a Bean Common Mosaic Virus (BCMV) infection after a Double Antibody Sandwich Enzyme Linked Immunosorbent Assay (DAS-ELISA) (see Supplementary Tables S1 and S2).

**Figure 1:**
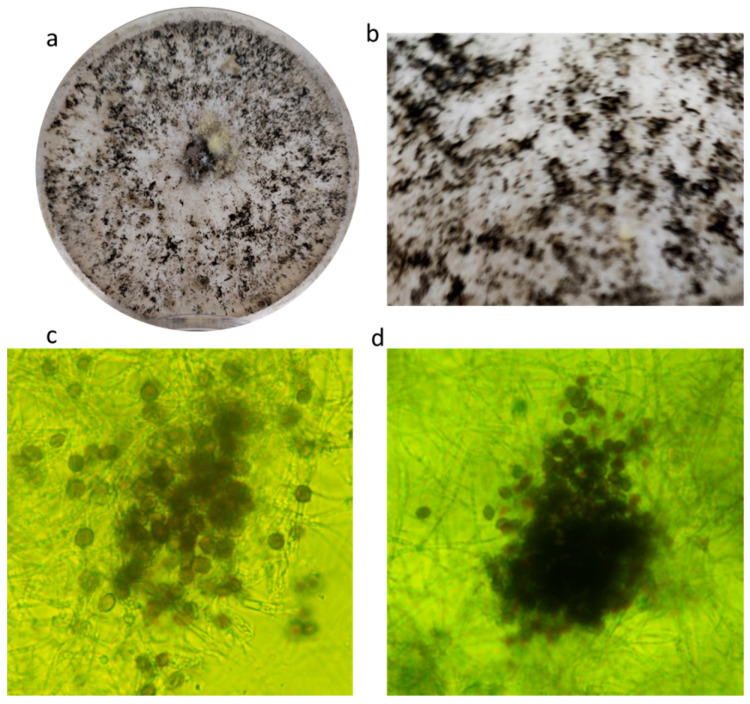
Morphology of *A. arundinis* CREA OF 1128.1: (**a**) colony on PDA; (**b**) details of black structures; (**c**) conidia of CREA OF 1128.1; (**d**) conidiogenous cells giving rise to conidia.

During the mycological screening on the *P. vulgaris* var. “Tondino di Castel di Sasso”, a white colony, not resembling known contaminants or key pathogens, developed on PDA plates. This strain, coded as CREA OF 1128.1, was selected for its ability to colonize the neighboring seeds and to inhibit their germination. The colony showed white and flat mycelium with black structures visible to the naked eye. Further analyses, performed with an optical microscope, highlighted the presence of globular-to-elliptic spores, brown or deep brown with an average diameter of 15 µm. Moreover, some agglomerates composed of conidiogenous cells were found, and they were arranged in variable shapes (Figure 1).

**Figure 2:**
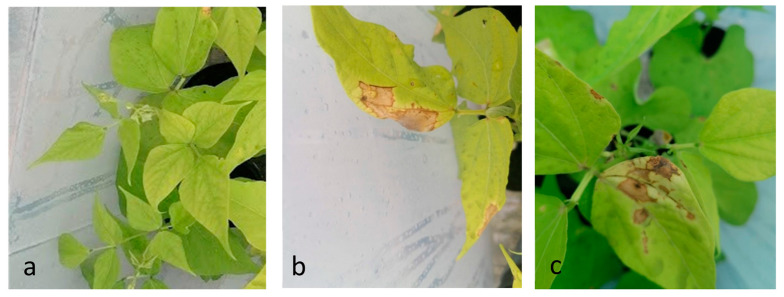
Common bean plants inoculated with *A. arundinis* CREA OF 1128.1: (**a**) non-inoculated control; (**b**,**c**) details of lesions on common bean leaves infected with *A. arundinis* CREA OF 1128.1.

The ITS1 and β-tubulin partial sequences (OQ132873 and OQ249627) allowed us to identify the strain as *Apiospora arundinis* (Corda) Pintos & P. Alvarado. The sequences (Table 2) were aligned using the BLASTn tool in the GenBank database, and the results showed a 98.39% identity with *A. arundinis* isolate F4P4Fg (OM959567) in the ITS locus and an identity of 99.40% with the strain MUCL1684 (AB220322) for the β-tubulin sequence, confirming that the strain CREA OF 1128.1 is *A. arundinis* (homotopic synonym: *Arthrinium arundinis*) [33]. The pathogenicity tests validated Koch’s postulates, since the strain was able to cause disease in the common bean. Indeed, dried lesions on the foliage, ranging from creamy white to light brown, were observed 21 days after inoculation on 87% of the inoculated plants (Figure 2).

**Figure 3:**
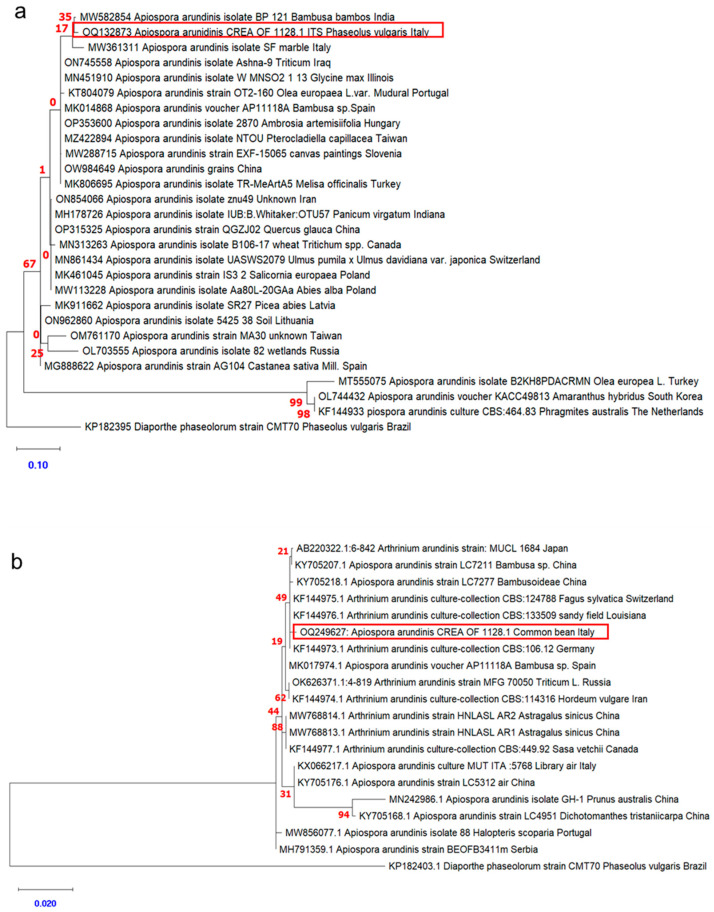
Phylogenetic analysis based on ITS (**a**) or β-tubulin (**b**) sequences of *Apiospora* spp. isolates. ITS1 and β-tubulin sequences of *D. phaseolorum* CMT70 from Brazil were used as an outgroup. The bootstrap tree inferred from 500 replicates is shown. The phylogenetic tree was inferred by using the maximum likelihood method and the Tamura–Nei model. The percentage of replicate trees in which the associated taxa clustered together in the bootstrap test is shown next to the branches. The tree is drawn to scale, with branch lengths measured in the number of substitutions per site (next to the branches). Red squares highlight the position of *A. arundins* CREA OF 1128.1. Numbers in red indicates the bootstraps for the nodes and the scale bar was reported in blue. Phylogenetic analyses were conducted in MEGA X.

A phylogenetic tree was built with MEGA X by aligning the sequences of CREA OF 1128.1 for both ITS and β-tubulin sequences, with *A. arundinis* sequences retrieved from GenBank (Figure 3). The results confirmed that the CREA OF 1128.1 isolate belonged to the species *A. arundinis*, as the novel isolate grouped in clusters containing most of the *A. arundinis* strains for both ITS and *TUB* genes. In the ITS phylogenic tree, CREA OF 1128.1 grouped with Italian strains SF (MW361311), collected from marble, thus suggesting a putative common ancestor based on the geographic location. On the other hand, CREA OF 1128.1 showed similarity also with an Indian isolate from bamboo, thus disavowing the hypothesis of geographic location. Moreover, the ITS sequences formed more than one clade, indicating slight variability among the strains. A similar result was observed in the phylogenetic tree obtained by aligning the β-tubulin sequences. Indeed, the CREA OF 1128.1 strain fit in a group with 49% bootstrap with strains isolated in Switzerland and Germany, but also with isolates from China and Louisiana, and this result did not support the hypothesis that strains with common geographical origin were more closely related from a phylogenetic point of view. Moreover, in the β-tubulin region, genetic variability was flattened with respect to ITS. In both phylogenetic trees, the *Diaporthe phaseolorum* (Cooke & Ellis) Sacc. CMT 70 strain was used as an outgroup.

The sample of “Tondino di Castel di Sasso” also resulted infected by the isolate CREA_OF_ 1128.3 of *Macrophomina phaseolina* (Tassi) Goid. The colony appeared flat and black, and abundant microsclerotia were macroscopically visible. The mycelium presented hyphae placed at 90° and the connection among the main hypha and the branches was characterized by the typical constrictions near the septa. The identity was confirmed by DNA extraction and subsequent amplification with the specific primer pairs MpCalF-MpCalR and MpTefF-MpTefR [32], reported in Table 3, that generated bands of 525 and 400 bp, as expected. The results of the mycological screening are summarized in Table S1.

Regarding the thirty-seven *P. vulgaris* accessions screened for the presence of key pathogenic bacteria, it is worth reporting that in the landrace “Dente di Morto” the presence of *Xanthomonas axonopodis* pv. *phaseoli* (Xap) and *X. fuscans* subsp. *fuscans* (Xff) was revealed in eight accessions propagated in 2021, while the bacteria were not detected in 2020. Moreover, Xap was detected in the accession “dei Signori”. As a consequence of these results, the infected seed lots were retired from the collection.

Pseudomonas-type colonies were detected in the landrace “Rosso di Acerra”, but the in vivo tests revealed that the isolates could not be recognized as *Pseudomonas savastanoi* pv. *syringae*.

Finally, the DAS-ELISA tests showed the presence of (a) the viruses CMV in seven common bean accessions (“Cannellino Bianco di Calitri”, “Lardari”, and five accessions of “Dente di Morto”), (b) the Bean Common Mosaic Virus (BCMV) in seven accessions (four accessions of “della Regina”, “Dente di Morto Acc. 2020”, “Butirro Acc. 2021”, and “Tabaccanti”), and (c) of mixed infection (CMV/BCMV) in two accessions (di “Prata Melizzano” and “Rosso di Acerra”) (Supplementary Table S2).

#### 2.2.2. Pea (*Pisum sativum* L.) and Lentils (*Lens culinaris* Medik.)

As expected, all eight pea accessions were found to be colonized by external contaminants such as *Alternaria* spp. (3% of contaminated seeds in one accession of “Centogiorni” and in one of “Santacroce”), *Penicillium* spp. and *Cladosporium* spp. (2% in “Santacroce Acc. T”), and *Chaetomium* spp. (44% in “Centogiorni Acc. 2021”). *Ascochyta pisi* Lib., a key pathogen, causing anthracnose in peas, was found in the pea accessions “Centogiorni from Pollenatrocchia” and “Santacroce accession M” (Table S1). The strains were coded as CREA OF 1122.4 and CREA OF 1122.5, respectively, and the sequence accession numbers obtained in GenBank were reported in Table 2. The BLASTn analyses conducted on the two strains revealed an identity of CREA OF 1122.4 (OQ148428) with the strain PA (MN653147) with a percentage of 99.16%, whereas the strain CREA OF 1122.5 (OQ148433) had an identity of 98.53% with the same GenBank accession, allowing us to identify the isolates as *A. pisi*.

No pathogenic fungi were detected from lentil seeds, whereas Fusarium-like colonies were isolated from diseased mother plants collected in the seed multiplication field (Table S1). The strain was named CREA OF 190.4 and identified as *Fusarium oxysporum* Schltdl., according to colony morphology and mycelium characteristics.

The isolate CREA OF 190.4 was analyzed by amplifying the ITS1 and *TEF 1*-α gene regions, using ITS1-4 and TEF_688/1251_ primer pairs. The PCR products were sequenced, and multiple alignments in GenBank were performed by using BLASTn. The isolates were finally identified as *F. oxysporum* f. sp. *medicaginis*, and the similarity was 100% for ITS (OQ134131) with the isolate XC02 (MT579855) and 100% with *F. oxysporum* f. sp. *medicaginis* isolate NRRL2254 (DQ837690.1) in the *TEF 1*-*α* locus (OQ249629) (Table 2).

**Figure 4:**
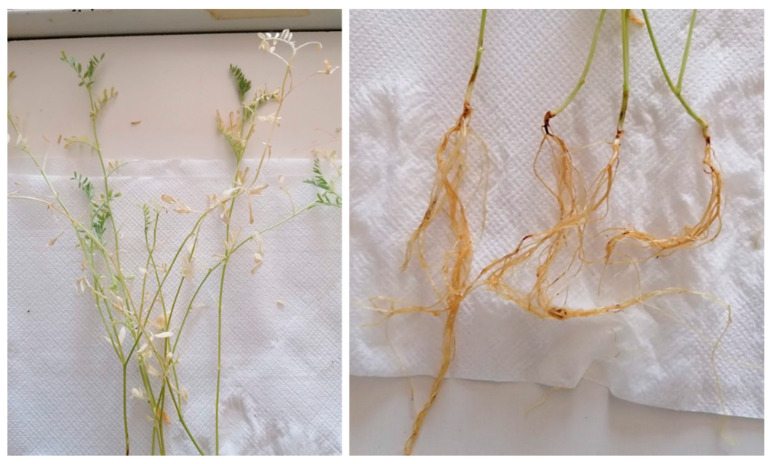
Symptoms caused by *F. oxysporum* CREA OF 190.4 on lentil seedlings. In the left panel the detail of foliage yellowing was reported and, on the right, the crown rot symptoms.

The test performed to verify the pathogenicity of *F. oxysporum* CREA OF 190.4 revealed a slight virulence of the isolate on lentil local variety “di Colliano” (Figure 4). The symptoms obtained in laboratory experiments were represented by necroses at collar level with a size ranging from 2 to 5 mm. After this symptom appearance, the leaves initially turned yellow and finally dropped. In our experimental conditions, 56% of the inoculated plants showed the described symptoms, while the non-inoculated control remained healthy, suggesting a putative partial resistance in the accession “di Colliano”. Moreover, no disease development was observed in inoculated plants of the *Medicago sativa* variety “Legend”, contrasting the molecular identification of *F. oxysporum* f. sp. *medicaginis*.

Finally, the virological and bacteriological analyses showed that pea accessions were not infected by CMV or *Pseudomonas syringae* pv. *pisi*.

#### 2.2.3. Broad Bean (*Vicia faba* L.) and Grass Pea (*Lathyrus cicera* L.)

In the case of the broad bean, in the accession “A Corna”, in addition to the common external contaminants, the key pathogen *Ascochyta fabae* Speg. (Teleomorph: *Didymella fabae*) was found, which is the causal agent of anthracnose [34] (Table S1). The identity of the strain, denominated CREA OF 1235.2, was confirmed by comparing ITS sequence OQ148403 with the nucleotide source in the NCBI database (a 96.16% identity was found with the isolate SA1, MN640757) (Table 2).

**Figure 5:**
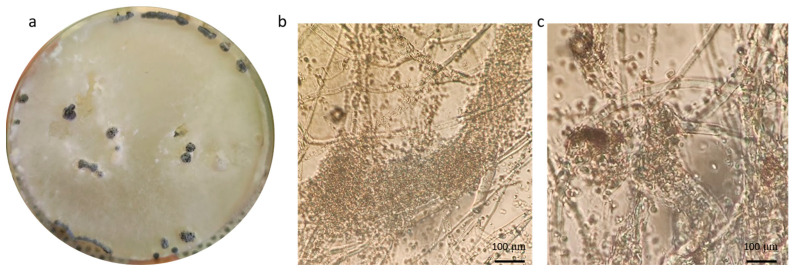
*S. sclerotiorum* CREA OF 1113.1 morphological features: (**a**) colony morphology on PDA and sclerotia after 15 days of growth; (**b**) ascopores and apothecium; (**c**) sclerotium development.

On the other hand, as a result of the mycological analyses of the broad bean local variety “Lunga (nocerino-sarnese) Acc. 2021”, the presence of a white colony with fluffy mycelium, able to grow on the seeds and inhibiting their germination, was observed in the isolation plates. Further morphological observation, revealing the presence of sclerotia, suggested that the colony CREA OF 1113.1 could be ascribed to *Sclerotinia sclerotiorum* (Lib.) de Bary, which is responsible for the white mold syndromes in several plants [35] (Table S1). The morphological analyses confirmed the colony features of *Sclerotinia* sp., showing (Figure 5) sclerotia with a mean diameter of 4.6 mm. Moreover, ascospores and apotecia with an average main diameter, respectively, of 9.0 µm and 5.2 mm were measured in the microscopic observation. The molecular analyses carried out using the ITS1-4 and Tef_728-986_ primer pairs confirmed the identification of CREA OF 1113.1 isolate as *S. sclerotiorum*. Indeed, BLASTn analyses showed that CREA OF 1113.1 isolate had a 99.09% identity with the isolate of *S. sclerotiorum* SCS18 (KY848721) in the ITS (OQ133464) region and 100% with the isolate ColPat-586 (MK089777) in the *TEF 1*-*α* sequence (OQ249628) (Table 2).

**Figure 6:**
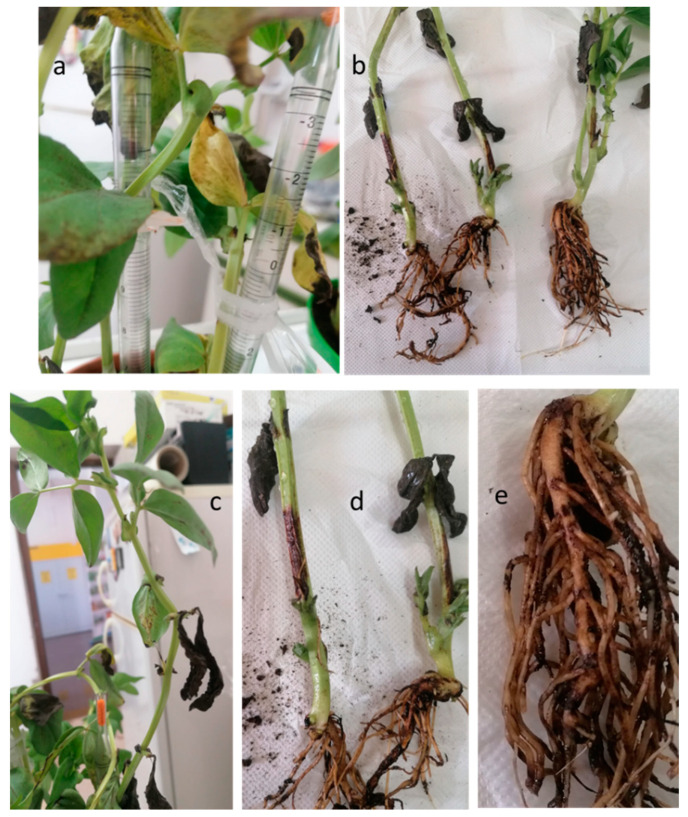
Symptoms on broad bean plants inoculated with *S. sclerotiorum* CREA OF 1113.1 twenty days post inoculation. (**a**) Chlorotic leaves; (**b**) stem rot spread from the inoculation point; (**c**) necrosis and desiccation; (**d**) detailed view of stem rot; (**e**) details of root rot.

Since this is the first time that the pathogen was reported in the broad bean in Italy, pathogenicity tests were performed to confirm Koch’s postulates. The symptoms of the disease (leaf chlorosis and stem rot) were visible after one week from the inoculation. At the end of the experimental time (20 days post inoculation), 100% of broad bean plants showed chlorosis and necrosis on their foliage along with serious stem rot around and far from the inoculation point (Figure 6). In addition, the roots appeared necrotic (Figure 6).

**Figure 7:**
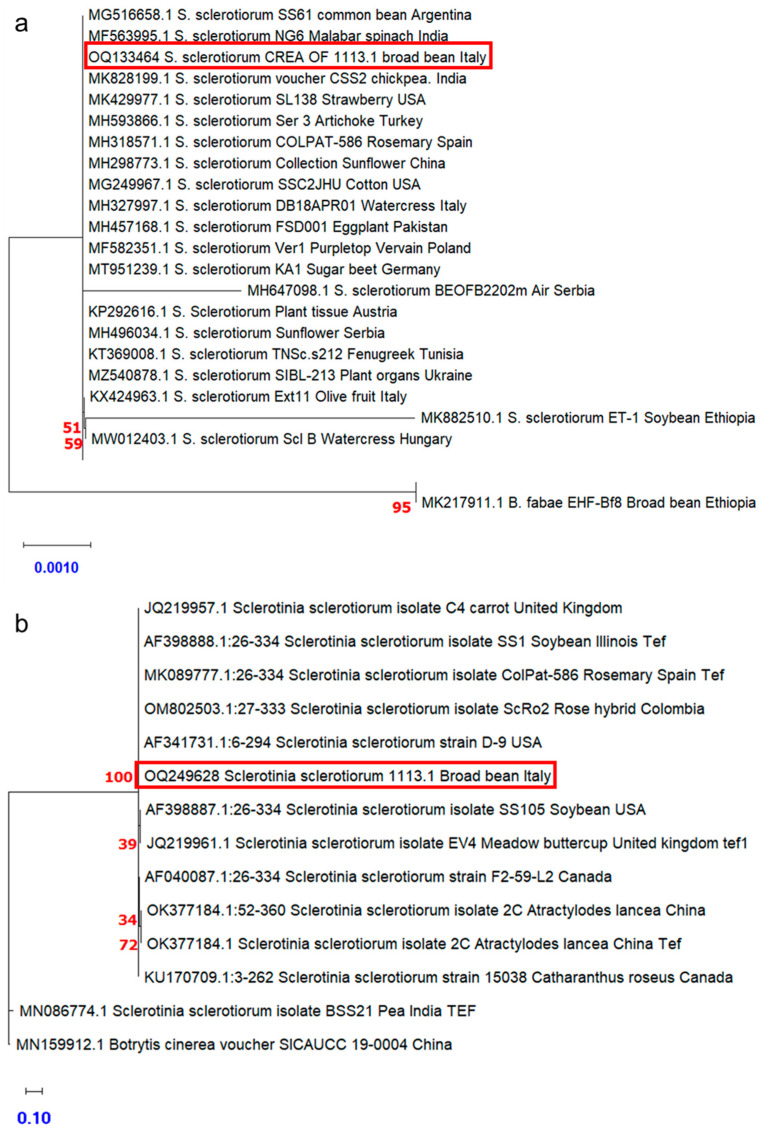
Dendrogram illustrating the evolutionary history of the isolated CREA OF 1113.1 inferred by ITS (**a**) and *TEF1*-*α* (**b**) DNA sequencing. For each taxon, the graph reports the GenBank accession number, the three-letter code pathogen species, the two-letter ISO country code, and the host plant species within vertical lines. The percentage of replicate trees in which the associated taxa clustered together in the bootstrap test (500 replicates) is shown. The tree is drawn to scale, with branch lengths in the same units as those of the evolutionary distances. Maximum Composite Likelihood method used to infer the phylogenetic tree. Red squares highlight the position of *S. sclerotiorum* CREA OF 1113.1. Numbers in red indicates the bootstraps for the nodes and the scale bar was reported in blue. Phylogenetic analyses were conducted in MEGA X.

The sequences obtained by the amplification of ITS, and *TEF1*-*α* regions by using the ITS1-4 and TEF_728/986_ primers pairs, were employed in a phylogenetic analysis making a comparison with sequences retrieved from the GenBank database, selected on the basis of plant host and geographic location. The strain *Botrytis fabae* EHF-Bf8 was used as a possible outgroup. The results obtained by analyzing the ITS region showed, as expected, that CREA OF 1113.1 formed a cluster with *S. sclerotium*, already reported in GenBank, with a significant bootstrap, indicating a limited genetic distance (Figure 7a). Similarly, the analyses of *TEF1*-*α* sequences (Figure 7b) showed that CREA OF 1128.1 grouped with 100% bootstrap support with accessions retrieved from GenBank obtained from different hosts, i.e., rosemary, rose, and soybean, originated in different geographic regions including the USA, Spain, and South America. Indeed, the observations suggested that, even if the sequences were selected based on different geographic locations and hosts, differences were not reflected in genetic variability.

**Figure 8:**
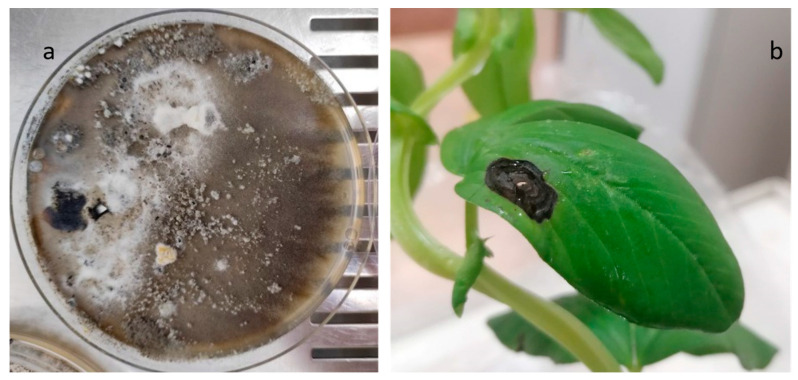
*S. vesicarium* CREA OF 1112.1 symptoms on inoculated broad bean “A sciabola” local variety: (**a**) colony morphology of CREA OF 1112.1 on PDA; (**b**) necrotic spots on broad bean leaves.

During the mycological surveillance, *Stemphylium vesicarium* (Wallr.) E.G. Simmons [36] was isolated from the broad bean local variety “A Sciabola Acc. 2021” (Table S1). The colony was coded as CREA OF 1112.1 and characterized by a grey-brownish colony with slightly fluffy mycelium. Under microscopic observation, in our experimental condition, no conidia were developed. Molecular analyses were performed through the amplification of the gene region ITS by using the ITS1-4 and the TEF_728-986_ primer pairs. The obtained sequences were aligned in BLASTn, and the isolate was identified as *S. vesicarium*. Indeed, CREA OF 1112.1 showed an identity of 99.26% with *S. vesicarium* isolate KACC48911 (MN871526) in the ITS (OQ266773) and 99.20% for the *TEF 1*-*α* locus (OQ249630) with the isolate NA51 (MH628128). To confirm the pathogenicity, CREA OF 1112.1 isolate was subjected to in vivo assay on *V. faba* “A Sciabola Acc. 2021” and, seven days after the inoculation, the compatible reaction was evidenced as typical necrotic spots on 52% of the leaves, with concentric circles, that became larger during the incubation time (Figure 8).

From wild grass pea local variety “Maracuoccio”, a strain (CREA OF 355.4) of *Stemphylium* sp. was also isolated (Table S1). The isolate was identified molecularly as *S. globuliferum* (Vestergr.) E.G. Simmons [37] by amplifying and sequencing the *TEF 1*-*α* region (Table 2); after a BLASTn analysis, the CREA OF 355.4 (OQ148588) strain showed a 99.81% identity with the strain of *S. globuliferum* FMB-NSIA-TA(S) (MG020726). Pathogenicity tests were performed but no symptoms were obtained on tested plants; thus, it was considered not pathogenic on the species of isolation.

**Figure 9:**
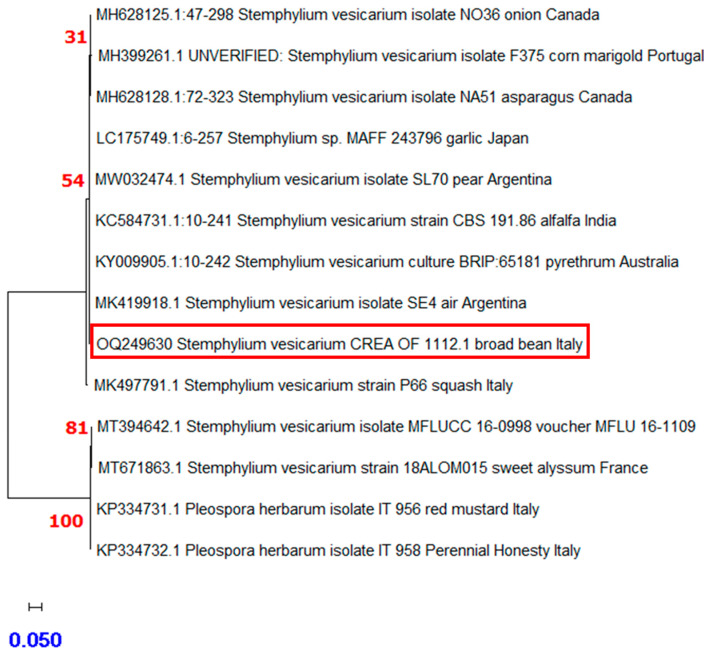
The evolutionary history inferred for *S. vesicarium* CREA OF 1112.1 based on *TEF 1*-*α* was performed by using the maximum likelihood method and the Tamura–Nei model. The tree with the highest log likelihood (−665.65) is shown. The percentage of trees in which the associated taxa clustered together is shown next to the branches. Initial tree(s) for the heuristic search were obtained automatically by applying the Neighbor-Join and BioNJ algorithms to a matrix of pairwise distances estimated using the Tamura–Nei model, and then selecting the topology with the superior log likelihood value. The tree is drawn to scale, with branch lengths measured in the number of substitutions per site (next to the branches). This analysis involved 13 nucleotide sequences. Codon positions included were 1st+2nd+3rd+Noncoding. There was a total of 733 positions in the final dataset. Red squares highlight the position of *S. vesicarium* CREA OF 1112.1. Numbers in red indicates the bootstraps for the nodes and the scale bar was reported in blue. Phylogenetic analyses were conducted in MEGA X.

A phylogenetic tree was built to confirm the identity of *S. vesicarium* CREA OF 1112.1. The analyses were conducted by aligning the *TEF 1*-*α* sequence (Table 2) obtained for the isolates with others retrieved from the GenBank database, considering different hosts and geographical origins. The results confirmed the identity of CREA OF 1112.1 as *S. vesicarium*. Indeed, the isolates grouped with previously described strains in clusters with 54% bootstrap, probably due to the differences in the sequence length. Moreover, from the phylogenetic tree, no correlation between the strains based on geographic origin and hosts could be inferred (Figure 9).

The broad bean results were negative in bacteriological and virological analyses for the detection of *P. syringae* and CMV, suggesting that, for these local varieties, the main concern was represented by mycological seed infections. Similarly, pathogenic bacteria were not detected in the grass pea (Table S2).

### 2.3. Brassicaceae

Among the *Brassicaceae* accessions tested, the most interesting results were obtained in *B. oleracea* var. *sabellica* “Torzella riccia”, on which the mycological screening revealed the presence of several *Alternaria* spp. which represent key fungal pathogens for this species (Table S1). The molecular analyses of the ITS1 partial sequences helped to identify the diverse isolates. In detail, CREA OF 1231.1, CREA OF 1231.4, CREA OF 1231.5 and CREA OF 1231.6, respectively related to the sequence OQ148383, OQ148398, OQ148397 and OQ148399, were identified as *Alternaria alternata* (Fries) Keissler. Moreover, the ITS sequence of CREA OF 1231.2 (OQ302538) aligned with *Alternaria infectoria* E.G. Simmons [38] sequence MK562061 with 98.93% identity, and CREA OF 1231.3 was identified as *Alternaria brassicicola* Schwein, Indeed the sequence OQ134394 showed 100% identity with the accession MW386633. The accession numbers of the sequences uploaded in GenBank are summarized in Table 2.

**Figure 10:**
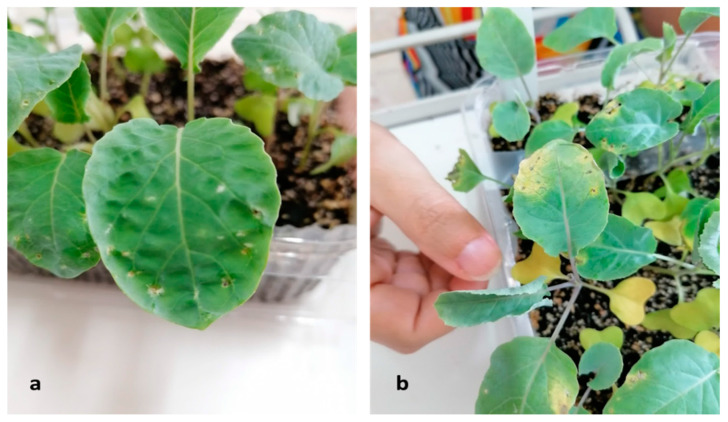
Infection of Xcc CREA OF 1231.20 in *B. oleracea* var. *botrytis*; (**a**) non-inoculate control; (**b**) symptomatic leaves.

In the same sample, the presence of *Xanthomonas campestris* pv. *campestris* (Xcc) (Pammel) Dowson was also evidenced (Table S2). The bacterial strains, coded as CREA OF 1231.20, CREA OF 1231.21, and CREA OF 1231.22, were isolated on mCS20ABN medium and resulted positive after PCR amplification according to the ISTA 7-019a protocol [23] using the DHL_153/154_ primer pair. Pathogenicity tests on *B. oleracea* variety “Palla di neve”, used as a positive control, confirmed the pathogenicity of the isolates, since the typical V-shaped necrotic lesions, surrounded by a chlorotic halo, were clearly observed on leaf veins (Figure 10).

## 3. Discussion

In the context of biodiversity preservation, this work evaluates health in seeds to be stored in germplasm banks and distributed to growers. The results of the screening carried out on Liliaceae, Fabaceace, and Brassicaceae accessions are discussed below.

To summarize, pathogenic fungi, bacteria, and viruses were present in 27%, 14%, and 27% of the accessions analyzed, respectively. In addition, novel pathogens were found in 3% of the examined accessions.

Regarding the Liliaceae crops, the analyses did not highlight pathogen contaminations in onion; while in garlic, *F. proliferatum* was detected. In Italy, this fungus was first reported, in the north, on garlic in 2011 [39] and subsequently was detected on the Welsh onion (*Allium fistulosum*) [40]. Therefore, the pathogen can be considered well established in Italy and is confirmed as a key pathogen for garlic. *F. proliferatum* is known to be one of the causal agents of Fusarium dry rot (FDR), a disease that asymptomatically affects *Allium* spp. in the field but that overall shows symptoms during postharvest. The asymptomatic behavior, associated with the vegetative propagation of the host, explains the dangerousness of the pathogen, which can survive for a long time in the clove tissues, compromising not only the storage but also the success of the new cultivations (where it is responsible for Fusarium basal rot) [41].

Regarding legumes, our study reported, for the first time in the common bean “Tondino di Castel di Sasso”, a pathogen identified as *A. arundinis*, which demonstrated its ability to infect the inoculated plants, causing necrotic spots on the leaves. The genus *Apiospora* is defined as cosmopolitan, being found in very different environments [42], and the reports of *A. arundinis* being a plant pathogen are very scarce. In Italy, it was found in the Florence Cathedral to cause the blackening of white marble [43] and it was isolated also from the environment in a library in Veneto [44]. The pathogenicity of this species was reported to cause different symptoms, such as a reddish stem in sugarcane and necrosis in bamboo in China, and necrotic lesions in rosemary in Iran [45,46,47]. This is the first report of *A. arundinis* on the common bean worldwide. It is speculated that the presence of this pathogen could be linked to climate change and to the temperature shift, which allows some microorganism to grow more easily with respect to previous decades. The same sample of “Tondino di Castel di Sasso” was contaminated by *M. phaseolina*, a necrotrophic seed-transmitted fungus, which caused charcoal rot and damping-off of the seedlings in the common bean and in over 500 other species [48]. In recent years, pathogen–pathogen interaction has been assumed to be the basis for the strengthening of disease expression, and several synergistic interactions are reported, also involving some other species of Botryosphaeraceae [49]. The control strategies against this pathogen are often frustrating because no resistance genes (inter alia, there is no applicable strategy in the case of landrace cultivation) or chemicals are available to counteract the disease. The persistence of microsclerotia as conservation organs and the polyphagous attitude of the pathogen nullify the effects of crop rotation. Conversely, an appropriate agronomical approach, mainly focused on the management of irrigation and favoring the preservation of beneficial microorganisms in the soil, can contribute to reduction of the disease incidence. It is clear that the use of certified seeds can limit the introduction of new inoculum in the fields.

As a result of the bacteriological analyses on *P. vulgaris*, the presence of *X. fuscans* subsp. *fuscans* (Xff) and *X. axonopodis* pv. *phaseoli* (Xap) was ascertained in the accession “dei Signori” and in most of the accessions of “Dente di morto”. Due to the high potential risk of transmission to plants and dissemination to the environment, the lots interested by the infection were removed from the collection. Moreover, diagnostic analyses were conducted on seed lots obtained in 2020 and the results indicated that Xap and Xff were not detected. This allowed for the recovery of healthy seed lots among those harvested in previous seasons. Lo Cantore et al. [50] reported that, already in 2002, Xap was found in southern Italy (Basilicata region) on the common bean landrace “Fagiolo di Sarconi”. In addition, in the same study the authors demonstrated a differential behavior of the plant materials tested against strains of Xap and Xff and concluded that the two landraces can be considered tolerant to the disease [50]. In 2014, the European Food Safety Authority (EFSA) determined that the risk associated with this pathogen ranged from moderate to high with several negative impacts on yield and quality [51]. Thereafter, the European and Mediterranean Plant Protection Organization (EPPO) highlighted its presence in all the European countries except for Finland, Sweden, and the United Kingdom [52], determining the shifting of the bacterium from quarantine pest to Regulated Non-Quarantine Pest (RNQP) [50].

Finally, BCMV was found in common bean and cowpea accessions [53]; in particular, on the former species, BCMV was sometimes associated with CMV. In Italy, mixed infection was documented in the Veneto region, starting from 2012 on the Lamon bean (*P. vulgaris*), an agricultural product obtained by the cultivation of four local varieties [54]. In addition, BCMV, as a single infective agent, was detected in 24% and 100% of the common bean and cowpea accessions tested, respectively. This high incidence in the cowpea is consistent with previous studies reporting that *V. unguiculata* is a major host of the virus [55]. In the case of viruses residing inside the embryo (such as CMV and BCMV), seed treatments with chemicals are often not effective and, in addition, resistance genes are present only in some genotypes. Several studies dealing with seed treatment with Plant Growth-Promoting Bacteria (PGPR) evidenced the PGPR’s effectiveness in improving the health of young plants and, consequently, their resistance to the early viral infections in *V. unguiculata* [56].

The analyses conducted on *P. sativum* revealed seed infection of *A. pisi* in the accessions “Centogiorni Acc. 2021” (from Pollenatrocchia) and “Santacroce Acc. M 2021”. *A. pisi* is one of the causal agents of anthracnose in the pea and it is one of the most studied pests in this species. The effect of seed infection was evaluated in Canada by Sivachandra et al. [57], who evidenced that the percentage of infection in seed lots (in the range 0.5–14.5%) was not related to seed germination or to the plant symptoms [57]. Conversely, the amount of rainfall after flowering seems to be associated with an increase in disease severity. Moreover, it was concluded that the transmission from the seeds to the plantlets did not significantly progress and that, in the case of *A. pisi*, the seeds represent mainly a way to store the pathogen, since it is present in all its parts [57]. The percentage of infection detected in this work on pea landraces is limited to 1%, thus indicating a phytosanitary status on this host that has to be monitored but that can be considered under control.

The seeds of lentil accessions analyzed in this study resulted noncontaminated by pathogenic fungi. Indeed, the major seedborne pathogens (*Ascochyta lentils*, *Botrytis cinerea*, and *F. oxysporum* f. sp. *lentils*) [25] resident on this species were not detected. Conversely, in the field located in Calitri (Salerno), pathogenic *F. oxysporum* was isolated from seed mother plants of local variety “di Colliano”. The isolates obtained in this work, after in vivo tests on a sample of “di Colliano” accession, caused slight crown rot and yellowing of leaves, but the molecular identification allowed us only to ascribe the strain to the *F. oxysporum* species. Usually, *F. oxysporum* f. sp. *lentils* is the causal agent of severe disease in lentils with symptoms of discoloration of the foliage and complete wilting of the plants [58]. This fungus was found in central Italy in 1999–2000 on a lentil local ecotype, but, according to the *TEF* sequencing done in our study, the strain found in Campania was identified as *F. o.* f. sp. *medicaginis* (Fome). Subsequently, this result was not confirmed by the inoculation on *M. sativa* variety “Legend”, whose susceptibility to *Fusarium* is unknown. On the other hand, the variety Yliki (Gaia seeds S.A., Greece), reported as susceptible to Fome [59], was not available for our research. Therefore, these results could be only considered as preliminary observations; further sequencing studies in different gene regions could provide more information about the taxonomy and the report of Fome in lentils.

The analysis of the phytosanitary status of broad bean seeds highlighted the presence of *A. fabae*, *S. sclerotiorum*, and *S. vesicarium* strains, and the pathogenicity tests carried out with these isolates demonstrated their ability to induce disease in plants. *A. fabae* (teleomorph *Didymella fabae*) is the causal agent of Ascochyta blight and was detected for the first time in 1991 in Cambridge (UK). According to the UK CAB International Distribution map [60], *D. fabae* was reported wherever the crop is cultivated (Italy included). The fungus attacks stems, leaves, fruit, and also the seeds, where it can be stored and transmitted to a new plant. Its spread in open fields can cause up to 95% yield losses in favorable conditions [61]. This makes an integrated disease control strategy, based on time of sowing and varietal choice, the most effective way to reduce the damage incidence in the crop [62]. Additionally, in this case, the use of pathogen-free seed is the first step for a successful cultivation. *S. sclerotiorum* is characterized by a wide host range, including several legumes in which it causes white mold, crown rot, and stem rot [35,63,64]. Moreover, *S. sclerotiorum* is known to produce severe economic and yield losses in the legume production systems [65,66], having a negative impact on seed quality [63]. In 2018, broad bean infection caused by *S. sclerotiorum* was reported in North Dakota with symptoms similar to those caused by the isolate CREA OF 1113.1 in laboratory tests, namely stem and root rot [67]. The CREA OF 1113.1 isolate was also involved in a phylogenetic analysis which confirmed the identity of the pathogen, since it grouped with strains previously isolated from different crops and geographical areas. The wide dissemination of this pathogen and the disease severity it causes suggest an urgent need for novel screenings that can identify and quickly counteract the infections [63]. In addition to *S. sclerotiorum*, in our study, strains of *S. vesicarium* were isolated from seeds of the broad bean and they showed the ability to produce the typical symptoms associated with *Stemphylium* disease. These observations are consistent with previous reports showing that *Stemphylium* spp. have a high variability in the host range, with some species characterized by a broad host range while others are very host-specific [37,68,69]. In this context, *S. botryosum*, *S. solani* and *S. vesicarium*, which are species commonly associated with legume diseases, were already reported in previous works [70,71,72,73,74,75,76]. Moreover, invasive *Stemphylium* infections on the broad bean were reported in 2016 in Australia [37], confirming the results obtained in this work. Our study represents the first report of the occurrence of *S. sclerotiorum* and *S. vesicarium* on the seeds of the broad bean in Italy.

Interestingly, following the mycological screening carried out during the monitoring, all the accessions of grass pea and wild grass pea (*L. sativum* and *L. cicera*) resulted noncolonized by fungal pathogens. The strain *S. globuliferum* CREA_OF_335.4 was isolated by wild grass pea (“Maracuoccio” landrace). Although the fungal species was previously recorded as a plant pathogen in *M. sativa* [77] and *Trifolium alexandrinum* L. [78], the strain isolated in this study resulted not pathogenic on the host in isolation. Thus, the “Maracuoccio” accession is judged as free from fungi that can compromise grass pea health, but a further characterization of CREA_OF_355.4 on alfalfa and clover could allow for the definition of its pathogenicity and the potential of its seeds as reservoir of the disease agent. The scientific literature on plant diseases in the grass pea is very scarce, probably due to its intrinsic resistance to plant pathogens. The *Lathyrus* spp. genome contains a pool of resistance genes able to counteract Ascochyta blight, *Erysiphe* spp., downy mildew (*Peronospora lathyri*-*palustris*), *Cercospora pisi sativae*, and *Uromyces* spp.. Moreover, wild Lathyrus species are also known to be resistant to broomrape (*Orobanche crenata*). According to a previous study, grass pea varieties can represent an alternative to traditional legume crops because of their agronomic traits and nutraceutical properties, and thanks to their resistance to biotic stress and drought and salinity tolerance [79].

The analyses conducted on Brassicaceae seeds highlighted that the most abundant isolates belonged to the genus *Alternaria*, including *A. alternata* and *A. brassicicola;* the latter is one of the commonly reported diseases on this plant species [80,81]. Moreover, the bacteriological analyses revealed the presence of *Xanthomonas campestris* pv. *campestris* (Xcc) on the local variety “Torzella riccia”. This is the first time that this bacterium has been reported on *Brassica oleracea* var. *sabellica*. Xcc represents one of the most common pathogens in the Brassicaceae family, including plants with high economic impact. It is endemic in central-southern Italy and it was diagnosed starting from the 1990s in broccoli landrace “Natalino”, in cauliflower and kohlrabi commercial varieties, and up to 2013 in the wild rocket [82,83]. As early as 1952, Xcc was reported as a bacterium primarily spread by seeds [84], and because of the ineffectiveness of chemicals and lack of resistance genes in local varieties, the importance of starting cultivation by using healthy propagation materials appears crucial [85].

In this work, the importance of seed health evaluation is evidenced. Many validated protocols are available to detect the key pathogens for which the seed is considered a pathway. No diagnostic procedures with minor economic impact are available for botanical species or for pathogens with limited diffusion, and for this reason, there is a risk that seed infection can escape phytosanitary control. When validated protocols were not available, the researchers had to balance the practical laboratory approach with the use of robust diagnostic procedures, within the limit of an appropriate budget, and taking into account the small number of seeds required for each accession to run the analyses. The ISF-regulated pest risk database, the EPPO Global Database https://www.seedtest.org/, accessed on 1 January 2020), and the ISTA Reference Pest List [17] represented powerful tools to plan the work. The use of molecular methods, such as ITS, *TEF*, and β-tubulin region sequencing, were confirmed to be effective to identify most of the isolates belonging to different fungal species, including pathogens reported for the first time on the hosts. In particular, in these cases, the molecular identification provided information to design the pathogenicity tests that are still considered the main tool to attribute the role of disease agent to a microorganism.

The seeds analyzed in this work showed the presence of common saprophytes that did not affect seed health, while key and novel pathogens were recorded in one third of the accessions. This confirmed the importance of full screening on seeds to be used as propagation materials, because it allows for the start of seed sanitation programs aiming to preserve and valorize the local varieties and their territories.

## 4. Materials and Methods

### 4.1. Sampling and Isolation of Fungal and Bacterial Strains from Seeds

Within the ABC project, landraces of Liliaceae (garlic and onion), Fabaceae (cowpea, common bean, pea, lentils, broad bean, and grass pea) and Brassicaceae (*B. oleracea* and *B. rapa*) were multiplied in the original area of cultivation (in situ) or in experimental farms (ex situ) with the aim of producing seeds to be stored in the germplasm bank. The list of the landraces taken into account in this study (in some cases landraces were represented by more than one accession), the type of analysis carried out on them, and the methods used are summarized in Table 1. The diagnostic studies were performed on 3 accessions of onion, 2 of garlic, 6 of *Brassica* spp., 5 of grass pea (representing two *Lathyrus* species), 37 of common bean (representing 27 landraces), 8 of pea (representing 2 landraces), 6 of broad bean (representing 3 landraces), and 3 of cowpea. For each species, the target pathogens (reported in Table 1) were chosen among the key pathogens listed in the ISF (International Seed Federation) regulated pest risk database [21] and for which the seed is considered a pathway. The pests never detected in Italian territory on the studied hosts, according to the EPPO Global Database, were excluded by the screening. For those vegetable species (garlic, grass pea, lentils, broad bean, and cowpea) not included in the ISF database, the criterion adopted to choose the target pests was based on the literature. For each accession, the phytosanitary status was evaluated on a representative sample of 300 seeds that was divided into three subsamples of 100 seeds, separately subjected to mycological, bacteriological, and virological analyses. In the case of garlic, whose commercial propagation material is represented by cloves, the sample consisted of 200 cloves, divided into 2 subsamples subjected to mycological and bacteriological analyses.

#### 4.1.1. Mycological Screening

In the case of mycological examination, the seeds were previously externally disinfected by a mild treatment with NaClO (0.5% active chloride) for 5–10 min. This treatment allowed us to avoid the overgrowth of *Rhizopus* sp. during the incubation without significantly compromising the isolation of pathogenic fungi. Then, the seeds were screened for the presence of the target microorganisms (Table 1) by plating them on potato dextrose agar (PDA, BD S.p.a., Milan, Italy) amended with 100 μg/mL chloramphenicol, 50 μg/mL streptomycin, and 50 μg/mL neomycin (Sigma-Aldrich, Milan, Italy), and incubating them for 10 days at 25 °C. Although this protocol was used to detect specific pathogens, the plating of seeds on such generic medium allowed us to also isolate other fungal species (pathogenic or not). This mycological examination protocol will be subsequently referred to as “mycological screening”. The number of seeds incubated in the 90 mm Petri dishes varied depending on the botanical species studied: nine seeds of common bean, cowpea, pea, and grass pea, six seeds of broad bean, twelve seeds of lentils, and sixteen seeds of brassica or onion. For garlic, 100 cloves were surface-sterilized with NaClO at 1% active chloride for 15 min, washed three times in sterile distilled water, and left to dry under a sterile hood. Then, the basal part of each clove was cut, put onto PDA, and incubated for 10 days at 25 °C. During the plate incubation, fungal colonies developed and, before their overgrowth, the isolates were transferred to fresh PDA plates. Finally, the colonies were observed for their morphological features and also used for the molecular and phylogenetic analyses (Section 4.5) and for the pathogenicity tests (Section 4.6). The mycological screening was carried out on all the species and accessions.

#### 4.1.2. Detection of Target Bacteria

The bacteriological analyses were carried out on garlic, common bean, broad bean, grass pea, cowpea, pea, and *Brassica* spp. seeds by adapting validated ISTA and ISHI-Veg protocols. In the case of the common bean and *Brassica* spp., the standard protocols ISTA 7-021, ISTA 7-023, and ISTA 7-019a (published in 2022) [23] were employed to detect Xap and Xff and Psp and Xcc, respectively. Some modifications were necessary to adapt the cited procedures because the seed samples available at the ABC gene bank were smaller than those recommended by ISTA protocols.

Briefly, 100 seeds were immersed in washing buffer (sterile saline plus Tween™ 20 (0.02% *v*/*v*) [23] and incubated at 4 °C overnight to allow the bacteria to be dispersed. Then, 1 mL of buffer was withdrawn and employed in a 10-fold dilution in the same buffer until dilution 10^−3^_,_ as reported in the “General methods” of the ISTA protocols. Briefly, each dilution was prepared by pipetting 0.5 mL from seed extract into a tube containing 4.5 mL of sterile diluent, which was then mixed prior to the next dilution step. A new sterile pipette was used for each dilution step. Then, 100 µL of diluted suspension and of undiluted extract were plated out, in triplicate, on the following selective media: Milk tween (MT) and Modified Sucrose Peptone medium (MSP) for the isolation of Xap, Xff, *P. s.* pv. *phaseolicola*, and *P. s.* pv. *syringae*. After five days, Pseudomonas and Xanthomonas-like colonies were purified on generic medium (Nutrient Agar, NA) to be submitted to further steps of identification and characterization.

Similarly, a modified ISTA 7-019a protocol was adopted to detect Xcc in *Brassica* spp. on the semi-selective media mCS20ABN and FS [23]. One hundred seeds were suspended in 1 mL of pre-chilled (2–4 °C) sterile saline plus Tween™ 20 (0.02% *v*/*v*) and shaken for 2.5 h at room temperature at 125 rpm. Then, the suspension was diluted until 10^−3^ (as reported above) and 100 µL was plated out on isolation media. After five days, Xanthomonas-like colonies were purified on NA and submitted to identification and characterization analyses.

Furthermore, the semiselective media KBBCA (King’s B Boric Acid medium) and SNAC (Sucrose NaCl medium) were used to select *P. syringae* pv. *pisi* according to the modified ISHI-Veg protocol [86]. One hundred seeds (1 gr of seeds per 2.5 mL solution) were macerated in sterile saline (0.85% NaCl) for 18 h. After incubation, the samples were shaken, and two serial 10-fold dilutions were prepared as reported above. Then, 100 µL of dilutions and undiluted seed extract were plated out on the specific selective media. MSP and King B media were used to attempt isolation of putative *Pseudomonas syringae* pathogenic pathovars from broad bean, grass pea, and cowpea. Maceration was performed in sterile saline (0.85% NaCl, 2.5 mL per 1 g seeds) and carried out overnight at 4 °C. Serial dilutions were obtained as previously described, and putative Pseudomonas colonies were purified on NA medium to be subjected to characterization analyses.

Finally, no isolation was performed on garlic cloves for the search of *Erwinia carotovora* subsp. *carotovora* because no rotted tissues were observed in any accession.

### 4.2. Virological Analyses

The virological analyses were conducted by grow-out assay, i.e., growing seedlings in controlled conditions, observing putative symptoms, and performing DAS-ELISA assays [87]. The tests were executed from symptomatic leaves and from asymptomatic bulk samples. The bulk samples were obtained by collecting five pieces of leaf tissue from five different plants and grinding them together 1/10 *w*/*v* in extraction buffer (Polyvinylpyrrolidone (PVP) 20 g, ovalbumin 2 g, sodium sulphite anhydrous 1.3 g in 1 L of phosphate buffered saline (PBS), pH 7.2, plus 10% Tween 20), as reported in the EPPO Standard 7/125 [87]. Noninfected and infected leaf tissues represented the NPC (Negative Processing Control, the baseline OD value for negative samples) and PPC (Positive Processing Control) as described in “Best practice for ELISA assay in seed health test”, published by ISF [86]. Blank control (buffers used in ELISA) was employed to evaluate the background optical density value in the absence of tissues. The samples and the controls were examined in duplicate. The assays were performed in Nunc Immunoplate maxiSorp and the results were read by measuring the absorbance at 405 nm by using the BioRad Microplate Reader, Model 550 (Bio Rad, Hercules, CA, USA). A sample was considered positive if the mean of two wells was higher than the double of the NPC. If the absorbance ranged between the NPC value and its double, the test was repeated.

DAS-ELISA testing was applied to verify putative infections of AMV, CMV, BCMV, and BCMNV in common bean, CMV and BCMV in cowpea, and CMV in pea and broad bean by using Bioreba ELISA Reagent sets (AMV Reagent set 480, Article code (Art.) Nr.: 140565; CMV Reagent set 960, Art. Nr.: 160662; BCMV Reagent set 480, Art. Nr.: 162065, and BCMNV Reagent set 480, Art. Nr.: 162165) with lyophilized positive control included (AMV Positive control component (C.) 140553; CMV Positive control C. 160653; BCMV Positive control C. 162053; and BCMNV Positive control C. 162153), following the manufacturer instructions (BIOREBA AG, Reinach, Switzerland). Besides the positive controls, the commercial sets included IgG, the secondary antibody conjugate to alkaline phosphatase, and the microtiter plates reported above.

### 4.3. Isolation from Symptomatic Mother Plants: The Case of Lentils

In late spring 2020, the accession of lentils “di Colliano”, multiplied in two open fields in Calitri (Salerno, Italy), showed, with an incidence of 40%, symptoms of wilting on the aerial part and crown and root blackening. Symptomatic radical tissues from five plants were washed in tap water, externally disinfected with 5% NaClO for 20 min, washed again in sterile distilled water, and dried under a hood. Fragments of 2–3 mm were cut from disinfected tissues and placed in PDA amended with antibiotics, as reported in Section 4.1. The plates were finally incubated at 25 °C in the dark for seven days. Fusarium-type colonies were transferred to fresh PDA plates and subjected to pathogenicity testing and molecular identification (Section 4.5 and Section 4.6).

### 4.4. Morphological Characterization of Fungal Colonies

The morphological characterization of fungal colonies was carried out macroscopically by observing the mycelium color and shape and the time required to overgrow the plate, whereas microscopic analyses were performed with an optical microscope, the Eclipse Nikon 90i with 10×, 20×, or 40× magnifications. Saprophyte fungi were recognized at the genus level according to the morphology features described by Champion [88]. The novel pathogens were characterized by colony morphology and, depending on the species, by microscopic specific features [89,90] such as: conidia shape and dimensions, hyphal morphology, mycelial color, presence/absence of sclerotia, presence/absence of sporangia, conidiophores, and conidiogenous cells. The dimensions of the specific structures were reported as a mean of twenty measurements.

### 4.5. Molecular Analyses and Phylogenesis

The fungal colonies preliminarily identified as belonging to putative pathogenic species were characterized by molecular analyses. First, spores were suspended in sterile water and monosporic cultures were obtained by plating the 10-fold dilution on PDA plates, with the aim of distinguishing pure single colonies. When fungi did not produce spores, monohyphal colonies were obtained by transplanting single mycelium tips grown on minimal medium (water agar). Monohyphal and monosporic cultures were then transferred in Potato Dextrose Broth (PDB, BD S.p.a., Milan, Italy) for 72 h at 25 °C and, finally, the grown mycelia were filtered using a sterile gauze. The samples were frozen at −80 °C, lyophilized and subjected to DNA extraction using the Genomic DNA isolation kit (Norgen, Biotek Corp., Thorold, ON, Canada) and following the manufacturer instructions. The PCR amplification was performed using different primer pairs based on the expected species as reported in Table 3. The couple ITS1 (5′-TCCGTAGGTGAACCTGCG–-3′)–ITS4 (5′-TCCTCCGTCTATTGATATGC-3′) was used to amplify the Internal transcribed spacer 1 region (ITS) from all the isolates studied. The primer pairs EF1–688F–EF1-1251R (5′-CGGTCACTTGATCTACAAGTGC-3′)/5′-CCTCGAACTCACCAGGTACCG-3′) and EF1–728F–EF1-968R (5′-CATCGAGAAGTTCGAGAAGG-3′/5′-TACTTGAAGGAACCCTTACC-3′) were employed to amplify the Translation elongation factor 1α (*TEF*-*1α*) sequences from *Fusarium* species and from all the other genera, respectively. Finally, the T1 (5′- TCCGTAGGTGAACCTGCGG–3′) and β-tub-2B (5′–ACCCTCAGTGTAGTGACCCTTGGC–3′) primer pair was used to amplify β-tubulin sequences of *Apiospora* sp.. For the PCR amplification, the Phusion™ High-Fidelity DNA Polymerase (2 U/µL) was employed according to manufacturer instructions and the PCR protocols were adapted for each primer pair. The PCR products were purified using the GeneJET PCR purification Kit (Thermofisher, Foster City, CA, USA) and sequenced by the Sanger method (BMR genomics, Padova, Italy). Sequences were trimmed and manually edited using Chromas Lite; they were identified using a nucleotide Basic Local Alignment Search Tool (BLAST) and then deposited in GenBank with the accession numbers reported in Table 2. Finally, the ITS, *TEF 1*-*α*, or *β*-*TUB* sequences were aligned using the default settings of Muscles, and then trimming was applied. The obtained alignments were employed to build phylogenetic trees using the max likelihood method and the Tamura–Nei model with 1000 bootstrap replicates using MEGA X.

Molecular identification of putative Xap, Xff, and Xcc was performed by using species-specific primer pairs, according to ISTA protocols mentioned above.

### 4.6. Pathogenicity Tests

The pathogenicity tests were carried out to verify Koch’s postulate only in the case of novel pathogen reports. Several inoculation protocols based on the pathosystems were employed, and the tests were performed in three replicates of ten plants, each belonging to the same local variety in which the isolates were found. The seed sample used for the pathogenicity assay was previously tested to ascertain the absence of pathogens. Negative controls were represented by plants treated with sterile water or agar, in the same conditions as those inoculated. Regarding *A. arundinis* CREA OF 1128.1, 10-day-old colonies were rinsed with 10 mL sterile deionized water and incubated for 10 min, to allow the spores to be dispersed; then, the spores were filtered, counted, and diluted in water to obtain a final suspension of 10^6^ spores/mL. The inoculation was performed in two replicates on ten common bean plants (“Tondino di Castel di Sasso” landrace), at the trifoliate stage, by spraying the suspension on both the lower and upper pages of the leaves to allow the inoculum to go through the stomata. The plants were incubated in RH up to 90% in plastic boxes in greenhouse conditions at 25 ± 2 °C, until symptoms were visible on the foliage. The non-inoculated control was obtained by spraying the leaves with distilled sterile water. The data were recorded as an average percentage of diseased plants in the two replications.

*S. sclerotiorum* CREA OF 1113.1 was inoculated on 3-week-old broad bean plants (local variety “Lunga”) by the V-shape method [91], which consists in cutting the plant stem and applying mycelium plugs of 10-day-old colonies on the wounds. Sterile mycelium was used as a negative control. Laboratory film was used to seal the inoculum and the experiments were conducted at 26 °C with a 12 h photoperiod in 90% humidity; the irrigations were performed manually when necessary. The disease consisted in leaf chlorosis visible after one week from the inoculation and developed into aerial wilting and final stem and leaf necrosis. After 20 days, the symptoms were recorded as black necrotic lesions on the stems and the leaves and root rotting. The number of plants showing the described symptoms was counted and reported as an average percentage in the two replicates.

In the case of *Stemphylium* isolates, 10-day-old colonies were rinsed with 10 mL sterile deionized water and incubated for 10 min, to allow the mycelium fragments to be dispersed; then, the obtained suspension was diluted to 10^6^ propagules/mL. The inoculation was performed on 3-week-old broad bean plants (“a Sciabola” landrace) by spraying the leaves at both the lower and upper page, to allow the inoculum to enter the stomata. Finally, the leaves were stinged with a sterile needle to facilitate the pathogen penetration. The plants were incubated in RH up to 90% in plastic boxes, in growth chamber conditions. Symptoms appeared 10 days after the inoculation and were visible on the foliage as brown spots. The non-inoculated control was obtained by spraying the leaves with distilled sterile water. The data were recorded as the percentage of spotted leaves in total on each plant and expressed as the mean in the sample.

*Fusarium* spp. colonies were inoculated in three-week-old lentil plants (“di Colliano” landrace) by dipping the roots in a 10^6^ conidia/mL suspension, obtained from a 10-day-old colony. Then, the plants were transplanted in a mixture of peat and sand (1:1 *v*/*v*) and grown in climatic chamber conditions at 26 °C with a 12 h photoperiod. Treatment with water was used as negative control, while the *Medicago sativa* variety “Legend” was used as a control to verify the pathogenicity of the strain that was molecularly identified as *F. o.* f. sp. *medicaginis*. The disease was recorded as the number of plants showing rot at the crown level with a consequent yellowing of the foliage; the data were expressed as an average percentage of diseased plants in the three replicates of six plants each.

The pathogenicity tests of bacterial isolates were carried out according to the ISTA7-023, 7-021 and 7-019a [23] protocols mentioned above. *P. vulgaris*, variety “Michelet”, was used as a susceptible control to establish the pathogenicity of *Xanthomonas*-like strains isolated from seed samples. According to the ISTA 7-021 protocol, the first trifoliate leaf was completely immersed in a 10^8^ CFU/mL bacterial suspension for 5 min. Then, the inoculated plants were incubated at 25 °C in a phytotron for 10 days. The symptoms of the disease were registered as typical polygonal chlorotic leaf spots surrounding leaf vein necrosis. Sometimes, necrotic streaks were also visible on the stems of inoculated plants. Plants treated with water represented the non-inoculated control. The strain of *Xanthomonas aonopodis* pv. *phaseoli*. LMG7455 was used as positive reference control.

The common bean variety “Michelet” was also used to test the pathogenicity of putative *Pseudomonas*. *savastanoi*. pv. *phaseolicola* isolate. Bean seeds were sown on sterile wet filter paper for 5 days and, at the beginning of the germination phase, the cotyledons were pricked with a sterile toothpick immersed in a 24-h-pure colony. Ten days after inoculation, the pathogenicity was assessed by observing the wounds on cotyledons. In the case of dried necrotic lesions, the strain was considered non-pathogenic. On the contrary, when hydropic halos surrounded the wounds, the strains were judged to be pathogenic. The strain of *P. s* pv. *phaseolicola* NCPPB 52.0 was used as a reference pathogenic strain.

*Brassica oleracea* var. *botrytis* variety “Palla di neve” was used as a susceptible control in the Xcc pathogenicity assays. In this case, sterile toothpicks were immersed in 24–48 pure Xcc-type colonies and used to prick the major veins of cauliflower leaves, at a point near the leaf edges. The plants were examined for the appearance of typical progressive V-shaped, yellow/necrotic lesions, with blackened veins, after 7–10 days at 25 °C. The strain PVCT 62.4 was used as a reference positive control. The pathogenicity tests for bacterial isolates were performed on six plants in three replicates.

## Figures and Tables

**Table 1:** List of accessions subjected to surveillance for seed health. Species, accession name, geographic area of cultivation, fungi, bacteria, and viruses searched.

Species	Accession	Geographic Area of Cultivation	Multiplication Field	Target Fungi (a)	Target Bacteria (b)	Target Viruses (c)	Analytical Methods (To Detect Pathogens (a, b, c))	Literature
*A. cepa*	Agostina	Agro acerrano-mariglianese (NA) ^1^	in situ	*Botrytis* spp. *Alternaria porri Stemphylium vesicarium*	/	/	(a) Mycological screening (agar plate method)	[20]
	di Alife (alifana)	Alife (CE) ^2^	ex situ					
	di Eremiti	Eremiti (SA) ^3^	in situ					
*A. sativum*	Bianco locale	Avellinese	in situ	*Fusarium* spp. *Botrytis* spp. *Macrophomina phaseolina Sclerotinia* spp.	*Erwinia carotovora* subsp. *carotovora*	/	(a) Mycological screening (agar plate method) (b) Isolation on Nutrient agar	[21,22]
	ecotipo locale di Caposele	Caposele (AV) ^4^	in situ					
*Brassica oleracea* var. *italica*	Broccolo del Vallo di Diano	Vallo di Diano (NA)	ex situ	*Alternaria brassicae*, *A. brassicicola*, *A. japonica*, *Phoma lingam Rizoctonia solani Sclerotinia* spp.	*Xanthomonas campestris* pv. *campestris and*	/	(a) Mycological screening (agar plate method) (b) ISTA 7-019a (dilution plating method)	[20,23]
	Broccolo dell’Olio	Agro nocerino-sarnese (SA)	ex situ					
	Broccolo di Natale	Agro acerrano-mariglianese (NA)	in situ					
	Broccolo San Pasquale	Agro acerrano-mariglianese (NA)	ex situ					
*Brassica oleracea* var. *sabellica*	Torzella riccia	Agro acerrano-mariglianese (NA)	ex situ					
*Brassica rapa* var. *rapa*	Catozza	Casertano, napoletano and Agro nocerino-sarnese (SA)	ex situ	*Alternaria brassicae*, *A. brassicicola*, *A. japonica*, *Phoma lingam Rizoctonia solani Sclerotinia* spp.	*Xanthomonas campestris* pv. *campestris*	/	(a) Mycological screening (agar plate method) (b) ISTA 7-019a (dilution plating method)	
*L. sativus*	Alta Irpinia	Avellinese	in situ	*Ascochyta* sp. *Fusarium* spp. *Botrytis* spp.	*Pseudomonas* spp.	/	(a) Mycological screening (agar plate method) (b) Dilution plating method	[24]
	di Calitri	Calitri (AV)	ex situ					
	di Colliano	Colliano (SA)	ex situ					
	di Montefalcone	Montefalcone (BN) ^5^	in situ					
*L. cicera*	Maracuocciolo/Maracuccia	Ascea/Camerota (SA)	ex situ	*Ascochyta* sp. *Fusarium* spp. *Botrytis* spp.	*Pseudomonas* spp.	/	(a) Mycological screening (agar plate method) (b) Dilution plating method	[24]
*L. culinaris*	di Colliano	Calitri (AV)	in situ	*Ascochyta* sp. *Fusarium* spp. *Botrytis* spp. *Sclerotinia* spp. *Rizoctonia solani*	/	/	(a) Isolation on PDA	[24,25]
*P. vulgaris*	Bianco di Villa Santa Croce	Villa di Santa Croce (CE)	ex situ	*Colletotrichum lindemuthianum Fusarium* spp. *Ascochyta phaseolorum Rizoctonia solani Macrophomina phaseolina Sclerotinia sclerotiorum Botrytis* spp.	*Pseudomonas savastanoi* pv. *phaseolicola Pseudomonas syringae* pv. *syringae Xanthomonas axonopodis* pv. *phaseoli*	AMV CMV BCMV BCMNV	(a) Mycological ^1^ screening (agar plate method) (b) ISTA 7-21; ISTA 7-023 (Dilution plating method) (c) DAS ELISA (EPPO Standard PM 7/125(1))	[23]
	Butirro Acc. 2021	Vico Equense (NA)	ex situ					
	Butirro Acc. 2020	Vico Equense (NA)	ex situ					
	Cannellino Sessantino dei 30 anni	Acerra (NA)	in situ					
	Cannellino Bianco di Calitri	Calitri (AV)	in situ					
	dei 7 anni	Visciano (NA)	in situ					
	dei Signori	Agro nocerino-sarnese (SA)	ex situ					
	della Regina	Valle d’ll’Angelo (SA)	ex situ					
	della Regina acc.2 2021 Bianco	Montano Antilia (SA)	ex situ					
	della Regina Acc.2 2021 Gandolfi	Montano Antilia (SA)	ex situ					
	della Regina Acc.2 2021 Mazzamauro	Montano Antilia (SA)	ex situ					
	della Regina Acc.2	San Lupo (BN)	ex situ					
	Della Regina di Gorga	Gorga (SA)	ex situ					
	della Regina	Montano Antilia (SA)	ex situ					
	Dente di Morto Acc. 2021	Agro acerrano-mariglianese (NA)	in situ					
	Dente di Morto Acc.1	Agro acerrano-mariglianese (NA)	in situ					
	Dente di Morto Acc.2	Agro acerrano-mariglianese (NA)	in situ					
	Dente di Morto Acc.3	Agro acerrano-mariglianese (NA)	in situ					
	Dente di Morto Acc.4	Agro acerrano-mariglianese (NA)	in situ					
	Dente di Morto Acc.5	Agro acerrano-mariglianese (NA)	in situ					
	Dente di Morto Acc.6	Agro acerrano-mariglianese (NA)	in situ					
	Dente di Morto Acc.7	Agro acerrano-mariglianese (NA)	in situ					
	Dente di Morto Acc. 2020	Agro acerrano-mariglianese (NA)	in situ					
	di Prata Melizzano	Prata Sanni–a–Melizzano (BN)	ex situ					
	di Volturara Irpina	Volturara Irpina (NA)	in situ					
	Fasulo a tubbettiello	Ruviano (CE)	ex situ					
	Giallo del Fortore	Alto Fortore (BN)	ex situ					
	Lardari	Agerola (NA)	in situ					
	Regina	Grottaminarda (AV)	in situ					
	Rosso di Acerra	Acerra (NA)	ex situ					
	Schiacciatello	Cellole (SA)	ex situ					
	Tabaccanti	Vallo di Diano (NA)	ex situ					
	Tondino Bianco di Calitri	Calitri (AV)	in situ					
	Tondino di Castel di Sasso Acc. 2022	Castel di Sasso (CE)	ex situ					
	Tondino di Villaricca	Villaricca (NA)	ex situ					
	Tondino di Villaricca	Villaricca (NA)	ex situ					
	Tondo Bianco	Sarno (SA)	ex situ					
*P. sativum*	Centogiorni 2021	Vesuvio/Agro nocerino-sarnese (NA)	ex situ	*Ascochyta* spp. *Macrophomina phaseolina Rizoctonia solani* *Fusarium* spp.	*Pseudomonas syringae* pv. *pisi*	CMV	(a) Mycological screening (agar plate method) (b) ISHI-Veg (Di-lution plating method) (c) DAS ELISA (EPPO Standard PM	[26]
	Centogiorni Acc. 2021	Acerra (NA)	ex situ					
	Centogiorni Acc. 2021	Pollenatrocchia (NA)	in situ					
	Centogiorni Somma Vesuviana Acc. 2021	Somma Vesuviana (NA)	in situ					
	Santacroce	Flegrea Area (NA)	in situ					
	Santacroce Acc. M 2021	Flegrea Area (NA)	ex situ					
	Santacroce Acc. N 2021	Flegrea Area (NA)	ex situ					
	Santacroce Acc. T 2021	Napoletano	ex situ					
*V. faba*	A sciabola Acc. 2021	Agro acerrano-mariglianese (NA)	in situ	*Ascochyta fabae Rizoctonia solani*	*Pseudomonas* spp.	CMV	(a) Mycological screening (agar plate method) (b) Dilution plating method (c) DAS ELISA (EPPO Standard PM 7/125(1))	[24] [27]
	Di Colliano	COLLIANO (SA)	ex situ					
	lunga	Somma Vesuviana (NA)	ex situ					
	lunga Acc. 2020	Nocerino-sarnese (NA)	ex situ					
	lunga Acc. 2021	Nocerino-sarnese (NA)	ex situ					
	A Corna	Agro acerrano-mariglianese (NA)	ex situ					
*V. unguiculata*	Corna dei Signori	Castel S. Giorgio (SA)	in situ	*Colletotrichum* sp., *Fusarium* spp. *Rizoctonia solani Macrophomina phaseolina Sclerotinia sclerotiorum*	*Pseudomonas* spp.	CMV, BCMV	(a) Mycological screening (agar plate method) (b) Dilution plating method (c) DAS ELISA (EPPO Standard PM 7/125(1))	[27]
	Corna dei signori (San Marzano)	San Marzano sul Sarno (SA)	ex situ					
	Fagiolino lungo San Marzano	San Marzano sul Sarno (SA)	in situ					

^1^: Naples; ^2^: Caserta, ^3^: Salerno; ^4^: Avellino; ^5^: Benevento.

**Table 2:** GenBank records of CREA OF isolates.

Accession Number	Locus	Strain Code	Species	Host/Species–Landrace
OQ280977	ITS	CREA OF 1115.1	*Alternaria alternata*	*Allium cepa*–“Agostina”
OQ132873	ITS	CREA OF 1128.1	*Apiospora arundinis*	*P. vulgaris*–“Tondino di Castel di Sasso”
OQ133464	ITS	CREA OF 1113.1	*Sclerotinia sclerotiorum*	*V.–faba–*“Lunga (nocerino-sarnese)
OQ134131	ITS	CREA OF 190.4	*Fusarium oxysporum*	*L. culinaris*–“di Colliano”
OQ134394	ITS	CREA OF 1231.3	*Alternaria brassicicola*	*B. oleracea* var. *sabellica*–“Torzella riccia”
OQ148383	ITS	CREA OF 1231.1	*Alternaria alternata*	*B. oleracea* var. *sabellica*–Torzella riccia”
OQ148397	ITS	CREA OF 1231.5	*Alternaria alternata*	*B. oleracea* var. *sabellica*– “Torzella riccia”
OQ148398	ITS	CREA OF 1231.4	*Alternaria alternata*	*B. oleracea* var. *sabellica*– “Torzella riccia”
OQ148399	ITS	CREA OF 1231.6	*Alternaria alternata*	*B. oleracea var. sabellica*– “Torzella riccia”
OQ148403	ITS	CREA OF 1235.2	*Ascochyta fabae*	*V. faba–*“A Corna”
OQ148428	ITS	CREA OF 1122.4	*Ascochyta pisi*	*P. sativum* –“Centogiorni (Pollenatrocchia)”
OQ148433	ITS	CREA OF 1122.5	*Ascochyta pisi*	*P. sativum–*“Santacroce Acc.M”
OQ148577	ITS	CREA OF 1119.1	*Fusarium proliferatum*	*A. sativum*–“Bianco locale”
OQ148588	ITS	CREA OF 355.4	*Stemphylium globuliferum*	*L. cicera*–“Maracuoccio”
OQ249627	β-tub	CREA OF 1128.1	*Apiospora arundinis*	*P. vulgaris*–“Tondino di Castel di Sasso”
OQ249628	*TEF 1*-*α*	CREA OF 1113.1	*Sclerotinia sclerotiorum*	*V. faba–*“Lunga (nocerino-sarnese)
OQ249629	*TEF 1*-*α*	CREA OF 190.4	*Fusarium oxysporum*	*L. culinaris*–“Di Colliano”
OQ249630	*TEF 1*-*α*	CREA OF 1112.1	*Stemphylium vesicarium*	*V. faba–“A Sciabola”*
OQ266773	ITS	CREA OF 1112.1	*Stemphylium vesicarium*	*Vicia faba* -*A sciabola*
OQ302538	ITS	CREA OF 1231.2	*Alternaria infectoria*	*B. oleracea* var. *sabellica*– “Torzella riccia”

**Table 3:** Primer pairs used to characterize the isolates.

Locus/Target	Definition	Primers	Sequence (5′-3′)	Reference	Isolates
EF-1α	Translation elongation factor 1α	EF1-728F	CATCGAGAAGTTCGAGAAGG	[28]	CREA OF 1113.1 CREA OF 1112.1
		EF1-968R	TACTTGAAGGAACCCTTACC		
		EF1-688F	CGGTCACTTGATCTACAAGTGC	[29]	CREA OF 190.4
		EF1-1251R	CCTCGAACTCACCAGGTACCG		
ITS1	Internal transcribing spacer 1	ITS1	TCCGTAGGTGAACCTGCGG	[30]	All
		ITS4	TCCTCCGTCTATTGATATGC		
β-tub	β-tubulin	T1	AACATGCGTGAGATTGTAAGT	[31]	CREA OF 1128.1
		β-tub-2B	ACCCTCAGTGTAGTGACCCTTGGC		
*Calmodulin*/*Macrophomina* spp.	Calmodulin	MpCalF	CAATCTCTTTCTCCCCTACAGGA	[32]	CREA OF 1128.3
		MpCalR	ACTGCGCAAAAGCGCCAGTAAAC		
		MsCalF	CAATGTCTTTCTCCACTGCAGGA		
		MsCalR	TACTGCGCCAAAGCGGCAGTAAA		
		MeCalF	AGCCCGCCTTGCTCCACCCTGTTCT		
		MeCalR	TGTATACTGCGCAAAAGCGGCAGT		
EF-1α	Translation elongation factor 1α	MpTefF	AAACACACTTTTCGCACTCCTGC-		
		MpTefR	TATGCTCGCAGAGAAGAACACGA		
		MsTefF	GGCACACTTTTCGCGCTTCTGTA		
		MsTefR	TGTGCTCGCTGGGAAGAACATGA		
		MeTefF	AAGCATACTTTTCGTGCTCCTGC		
		MeTefR	AAAGGAACATGAGTGGCCAAAAA		
Xap/Xff		p7X4c	GGCAACACCCGATCCCTAAACAGG	[23]	CREA OF 522, 539, 540, 541, 542, 543, 544, 545, 546
		p7X4e	CGCCGGAAGCACGATCCTCGAAG		
Xcc		DLH120	CCGTAGCACTTAGTGCAATG	[23]	CREA OF 1231.20, 1231.21, 1231.22
		DLH125	GCATTTCCATCGGTCACGATTG		

## Data Availability

All data included in the main text and Supplementary Materials.

## Supplementary Materials

The following supporting information can be downloaded at: https://www.mdpi.com/article/10.3390/plants12040812/s1. Table S1: Results of mycological screening; Table S2: Overview of bacteriological and virological results.
