# Supplementary material for: Surveillance of Landraces’ Seed Health in South Italy and New Evidence on Crop Diseases
Source: Plants (Basel). 2023 Feb 11;12(4):812. doi: 10.3390/plants12040812 (PMC9959537; doi:10.3390/plants12040812)
Supplement: Supplementary file 1 [file plants-12-00812-s001.zip › plants-2166844-supplementary.pdf]

Table S1. Results of mycological screening.

**Species**	**Accession**	***Fungi***
A. cepa	Agostina	*2% Alternaria* spp.
	di Alife (alifana)	Not detected
	di Eremiti	*1% Aspergillus niger*
A. sativum	Bianco locale	*Not detected*
	ecotipo locale di Caposele	*1% Fusarium proliferatum*
Brassica oleracea var. *italica*	Broccolo del Vallo di Diano	*17% Penicillium sp.*<br>*2% Aspergillus spp.*
	Broccolo dell'Olio	*2% Alternaria spp.*<br>*1%Fusarium spp.*
	Broccolo di Natale	*Not detected*
	Broccolo San Pasquale	*Not detected*
Brassica oleracea var. sabellica	Torzella riccia	*6% Alternaria spp. (Alternaria alternata, Alternaria infectoria, Alternaria brassicicola)*
Brassica rapa var. *rapa*	Catozza	*100% Alternaria spp.*
L. sativus	Alta Irpinia	*3% Penicillium*
	di Calitri	*Not detected*
	di Colliano	*Not detected*
	di Montefalcone	*6% Rhizopus*
L. cicera	Maracuocciolo/Maracucc ia	*3% Alternaria spp., 1% Stemphylium globuliferum*
Lens culinaris	di Colliano	*Detection of Fusarium oxysporum in symptomatic plants*
P. vulgaris	Tondino di Castel di Sasso Acc. 2022	*1 % Macrophomina phaseolina*<br>*1% Apiospora arundinis*
P. sativum	Centogiorni 2021	*44% Chaetomium spp.*<br>*3% Alternaria spp.*
	Centogiorni Acc. 2021 (Pollenatrocchia)	*2% Ascochyta pisi*
	Centogiorni Acc. 2021 ex situ	*Not detected*

Species	Accession	*Fungi*
	Centogiorni Somma Vesuviana Acc. 2021	*Not detected*
	Santacroce	*Not detected*
	Santacroce Acc. M 2021	*2% Ascochyta pisi*<br>*3% Alternaria alternata*
	Santacroce Acc. N 2021	*Not detected*
	Santacroce Acc. T 2021	*2% Cladosporium sp.; 1% Penicillium sp.*
V. faba	A Corna	*3% Ascochyta fabae*
	A sciabola Acc. 2021	*1% Chaetomium*<br>*1% Stemphylium vesicarium*<br>*1% Alternaria alternata*
	lunga di Somma Vesuviana	*Not detected*
	lunga Acc. 2020 nocerino-sarnese	*Not detected*
	lunga Acc. 2021 nocerino-sarnese	*1% Sclerotinia sclerotiorum*<br>*1% Alternaria alternata*
V. unguiculata	Corna dei signori (San Marzano)	*2% Alternaria spp.*
	Fagiolino lungo San Marzano	*Not detected*
	Corna dei Signori (Castel S. Giorgio)	*1% Chaetomium*

Table S2. Overview of bacteriological and virological results

Species	Accession	Geographic area of cultivation	*Bacteria*	Viruses
A. sativum	Bianco locale	Avellinese	*Not detected*	/
	ecotipo locale di Caposele	Caposele (AV)		
Brassica oleracea var. *italica*	Broccolo del Vallo di Diano	Vallo di Diano (NA)	*Not detected*	/
	Broccolo dell'Olio	Agro nocerino-sarnese (Salerno)	*Not detected*	
	Broccolo di Natale	Agro acerrano-mariglianese (NA)	*Not detected*	
	Broccolo San Pasquale	Agro acerrano-mariglianese (NA)	*Not detected*	
Brassica oleracea var. sabellica	Torzella riccia	Agro acerrano-mariglianese (NA)	*Xanthomonas campestris pv. campestris*	

Species	Accession	Geographic area of cultivation	*Bacteria*	Viruses
Brassica rapa var. *rapa*	Catozza	Casertano, napoletano and Agro nocerino-sarnese (SA)	*Not detected*	/
L. sativus	Alta Irpinia	Avellinese	*Not detected*	/
	di Calitri	Calitri (AV)	*Not detected*	
	di Colliano	Colliano (SA)	*Not detected*	
	di Montefalcone	Montefalcone (BN)	*Not detected*	
Phaseolus vulgaris	Bianco di Villa Santa Croce	Villa di Santa Croce (CE)	*Not detected*	*Not detected*
	Butirro Acc. 2021	Vico Equense (NA)	*Not detected*	BCMV
	Butirro Acc. 2020	Vico Equense (NA)	*Not detected*	*Not detected*
	Cannellino Sessantino dei 30 anni	Acerra (NA)	*Not detected*	*Not detected*
	Cannellino Bianco di Calitri	Calitri (AV)	*Not detected*	CMV
	dei 7 anni	Visciano (NA)	*Not detected*	*Not detected*
	dei Signori	Agro nocerino-sarnese (SA)	*Xanthomonas axonopodis pv. phaseoli/var fuscans*	*Not detected*
	della Regina	Valle dell'Angelo (SA)	*Not detected*	BCMV
	della Regina acc .2 2021 Bianco	Montano Antilia (SA)	*Not detected*	*Not detected*
	della Regina acc. 2 2021 Gandolfi	Montano Antilia (SA)	*Not detected*	BCMV
	della Regina Acc. 2 2021 Mazzamauro	Montano Antilia (SA)	*Not detected*	BCMV
	della Regina Acc.2	San Lupo (BN)	*Not detected*	BCMV
	Della Regina di Gorga	Gorga (SA)	*Not detected*	*Not detected*
	della Regina	Montano Antilia (SA)	*Not detected*	*Not detected*
	Dente di Morto Acc. 2021	Agro acerrano-mariglianese (NA)	*Xanthomonas axonopodis pv. phaseoli/var fuscans*	CMV
	Dente di Morto Acc.1	Agro acerrano-mariglianese (NA)	*Xanthomonas axonopodis pv. phaseoli/var fuscans*	*Not detected*
	Dente di Morto Acc.2	Agro acerrano-mariglianese (NA)	*Xanthomonas axonopodis pv. phaseoli/var fuscans*	CMV
	Dente di Morto Acc.3	Agro acerrano-mariglianese (NA)	*Xanthomonas axonopodis pv. phaseoli/var fuscans*	*Not detected*

Species	Accession	Geographic area of cultivation	*Bacteria*	Viruses
	Dente di Morto Acc.4	Agro acerrano-mariglianese (NA)	*Xanthomonas axonopodis pv. phaseoli/var fuscans*	*Not detected*
	Dente di Morto Acc.5	Agro acerrano-mariglianese (NA)	*Xanthomonas axonopodis pv. phaseoli/var fuscans*	CMV
	Dente di Morto Acc.6	Agro acerrano-mariglianese (NA)	*Xanthomonas axonopodis pv. phaseoli/var fuscans*	CMV
	Dente di Morto Acc.7	Agro acerrano-mariglianese (NA)	*Xanthomonas axonopodis pv. phaseoli/var fuscans*	CMV
	Dente di Morto Acc. 2020	Agro acerrano-mariglianese (NA)	*Not detected*	BCMV
	di Prata Melizzano	Prata Sannita - Melizzano (BN)	*Not detected*	CMV-BCMV
	di Volturara Irpina	Volturara Irpina (NA)	*Not detected*	*Not detected*
	Fasulo a tubbettiello	Ruviano (CE)	*Not detected*	*Not detected*
	Giallo del Fortore	Alto Fortore (BN)	*Not detected*	*Not detected*
	Lardari	Agerola (NA)	*Not detected*	CMV
	Regina	Grottaminarda (AV)	*Not detected*	*Not detected*
	Rosso di Acerra	Acerra (NA)	*Not detected*	CMV-BCMV
	Schiacciatello	Cellole (SA)	*Not detected*	*Not detected*
	Tabaccanti	Vallo di Diano (NA)	*Not detected*	BCMV
	Tondino Bianco di Calitri	Calitri (AV)	*Not detected*	*Not detected*
	Tondino di Castel di Sasso Acc. 2022	Castel di Sasso (CE)	*Not detected*	*Not detected*
	Tondino di Villaricca	Villaricca (NA)	*Not detected*	*Not detected*
	Tondino di Villaricca	Villaricca (NA)	*Not detected*	*Not detected*
	Tondo Bianco	Sarno (SA)	*Not detected*	*Not detected*
P. sativum	Centogiorni 2021	Vesuvio / Agro nocerino-sarnese (NA)	*Not detected*	*Not detected*
	Centogiorni Acc. 2021	Acerra (NA)	*Not detected*	*Not detected*
	Centogiorni Acc. 2021	Pollenatrocchia (NA)	*Not detected*	*Not detected*
	Centogiorni Somma Vesuviana Acc. 2021	Somma Vesuviana (NA)	*Not detected*	*Not detected*

**Species**	**Accession**	**Geographic area of cultivation**	***Bacteria***	**Viruses**
	Santacroce	Flegrea Area (NA)	*Not detected*	*Not detected*
	Santacroce Acc. M 2021	Flegrea Area (NA)	*Not detected*	*Not detected*
	Santacroce Acc. N 2021	Flegrea Area (NA)	*Not detected*	*Not detected*
	Santacroce Acc. T 2021	Napoletano	*Not detected*	*Not detected*
V. faba	A sciabola Acc. 2021	Agro acerrano-mariglianese (NA)	*Not detected*	*Not detected*
	A Corna	Agronocerono-mariglianese	*Not detected*	*Not detected*
	lunga	Somma Vesuviana (NA)	*Not detected*	*Not detected*
	lunga Acc. 2020	Nocerino-sarnese (NA)	*Not detected*	*Not detected*
	lunga Acc. 2021	Nocerino-sarnese (NA)	*Not detected*	*Not detected*
Vigna unguiculata	Corna dei signori (San Marzano)	San Marzano sul Sarno (SA)	*Not detected*	BCMV
	Fagiolino lungo San Marzano	San Marzano sul Sarno (SA)	*Not detected*	BCMV
	Corna dei Signori	Castel S. Giorgio (SA)	*Not detected*	BCMV

Legend: / = not tested
